# Cyclodextrin Complexation Improves the Solubility and Caco-2 Permeability of Chrysin

**DOI:** 10.3390/ma13163618

**Published:** 2020-08-16

**Authors:** Ferenc Fenyvesi, Thi Le Phuong Nguyen, Ádám Haimhoffer, Ágnes Rusznyák, Gábor Vasvári, Ildikó Bácskay, Miklós Vecsernyés, Simona-Rebeca Ignat, Sorina Dinescu, Marieta Costache, Alina Ciceu, Anca Hermenean, Judit Váradi

**Affiliations:** 1 Department of Pharmaceutical Technology, Faculty of Pharmacy, University of Debrecen, Nagyerdei St. 98, H-4032 Debrecen, Hungary; fenyvesi.ferenc@pharm.unideb.hu (F.F.); nguyen.thi.le.phuong@pharm.unideb.hu (T.L.P.N.); haimhoffer.adam@pharm.unideb.hu (Á.H.); rusznyak.agnes@pharm.unideb.hu (Á.R.); vasvari.gabor@pharm.unideb.hu (G.V.); bacskay.ildiko@pharm.unideb.hu (I.B.); vecsernyes.miklos@pharm.unideb.hu (M.V.); 2 Doctoral School of Pharmaceutical Sciences, University of Debrecen, Nagyerdei St. 98, H-4032 Debrecen, Hungary; 3 Department of Biochemistry and Molecular Biology, Faculty of Biology, University of Bucharest, Splaiul Independentei 91-95, 050095 Bucharest, Romania; simona.ignat@unibuc.ro (S.-R.I.); sorina.dinescu@bio.unibuc.ro (S.D.); marieta.costache@bio.unibuc.ro (M.C.); alina_ciceu@yahoo.com (A.C.); anca.hermenean@gmail.com (A.H.); 4 Research Institute of the University of Bucharest, Splaiul Independentei 91-95, 050095 Bucharest, Romania; 5 “Aurel Ardelean” Institute of Life Sciences, Vasile Goldis Western University of Arad, 86 Revolutiei, 310414 Arad, Romania

**Keywords:** chrysin, 2-hydroxypropyl-beta-cyclodextrin, random methyl-beta-cyclodextrin, Caco-2 permeability, phase-solubility

## Abstract

Chrysin is a bioflavonoid that can be found in natural products such as honey and propolis, and it possesses several biological effects such as antioxidant, anti-inflammatory, and anti-cancer activity. However, it is poorly soluble in water, and its bioavailability is limited. The aim of this research is to investigate the chrysin solubilization capacity of different β-cylcodextrin derivatives and compare their biological activities. Chrysin was complexed with β-cyclodextrin (βCD), hydroxypropyl-β-, (HPBCD) sulfobutylether-β-, (SBECD), and randomly-methylated-β-cyclodextrin (RAMEB) by the lyophilization method in 1:1 and 1:2 molar ratios. The solubilities of the chrysin–cyclodextrin complexes were tested, and the solubilization abilities of cyclodextrins were studied by phase solubility experiments. The cytotoxicity of the complexes was measured by the MTT method, and the permeability enhancement was tested on Caco-2 monolayers. The solubility study showed that the complexes formed with RAMEB had the highest solubility in water. The phase solubility experiments confirmed the strongest interaction between RAMEB and chrysin. In the viability test, none of the complexes showed cytotoxicity up to 100 µM concentration. The permeability study revealed that both at 1:1 and 1:2 ratios, the RAMEB complexes were the most effective to enhance chrysin permeability through the Caco-2 monolayers. In conclusion, cyclodextrins, especially RAMEB, are suitable for improving chrysin solubility and absorption.

## 1. Introduction

**Figure 1:**
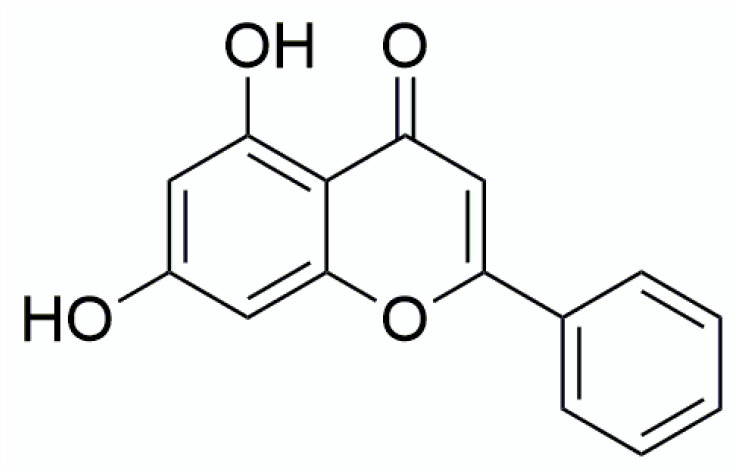
The chemical structure of chrysin.

Chrysin (5,7-dihydroxylflavone) belongs to the flavonoids class of polyphenolic compounds (Figure 1). The chemical structure of chrysin differs from other flavonoids in lacking oxygenation in the B-ring and oxygenation at C3 of the C-ring, as well as the presence of a C2–C3 double bond in ring C. Due to this lack of oxygenation of the B and C ring, the chemical features of chrysin are related to their pharmacological activities. It possesses antioxidant activity [1] and also other biological effects including anti-inflammatory and anti-cancer [2,3,4]. Chrysin occurs mostly in plants such as the Indian trumpet tree (Oroxylum indicum) and blue passion flower (Passiflora caerulae, Passiflora incarnata), and it can be found in honey and propolis [5]. Even though chrysin is quite polar due to its phenolic structure, its aqueous solubility is low, which is a problem for bioavailability. It was recorded that the concentration of chrysin in plasma after oral administration of a 400 mg dose to healthy human volunteers was low: only 12–64 nM [6]. This kind of characteristic limits pharmaceutical applications of chrysin despite the fact that it is a promising agent with many health and nutritional benefits. Pharmaceutical formulators apply several techniques to overcome the insolubility without diminishing the drug’s potency. Some of those techniques include micronization, nanosuspension, derivatization, complexation, salt formation, or the use of solubilizers, surfactants, and so on. Among all of the mentioned techniques, complexation has been put to use specifically to modify the solubility, dissolution rate, and bioavailability of lipophilic drugs. 

Cyclodextrins (CDs) have been used as pharmaceutical excipients for over 100 years, and in the past 30 years, their application is widespread as a result of improvements in its manufacture as well as production costs [7]. Cyclodextrins are cyclic oligosaccharides composed of (a-1-4)-linked α-D glucopyranose units arranged in a donut-shaped ring. They have lipophilic inner cavity and a hydrophilic outer surface, so this aspect makes them soluble in water, but the cavity provides a hydrophobic habitat. Their structure significantly influences the biocompatibility; we previously showed that there is a relationship between the cytotoxicity and cholesterol complexation abilities [8]. The hydrophilic derivatives of β-cyclodextrin such as hydroxypropyl-β-cylcodextrin (HPBCD) and sulfobutylether-β-cylcodextrin (SBCD) are not toxic at low to moderate doses, and they can be parenterally administered [9,10]. Randomly-methylated-β-cyclodextrin (RAMEB) is a lipophilic derivative, although it is water soluble and good at binding capacity [11]. Cyclodextrins are able to form complexes with drug molecules when they are in aqueous solution, whereby β-cyclodextrins generally form complexes with aromatic and heterocyclic molecules. As a result, the solubility of the complexed drug increases, which is also reflected in the surface tension changes of the system [12]. In this present work, we studied the solubilization efficacy capacity of four different derivatives of β-cyclodextrins with chrysin and determined by phase solubility test. At different ratios of different complexes, we aimed to test how cyclodextrins improve the solubility of chrysin, examining whether it would depend on the types of drug carrier and/or the amount of it. Furthermore, the bioactive properties of different complexes were also investigated. There are some contradictory studies using the Caco-2 adenocarcinoma cell line which state that chrysin has favorable membrane transport properties [13] or that it is poorly transported via the passive diffusion pathway in Caco-2 cells [14]. Nevertheless, the chrysin permeability-improving abilities of cyclodextrins on Caco-2 monolayers have not been studied yet. Our aim was to test the complexation and solubilization of chrysin by different cyclodextrin derivatives and study the improvement of chrysin permeation by cyclodextrins through the Caco-2 monolayers.

## 2. Materials and Methods 

### 2.1. Materials

(2-hydroxypropyl)-β-cyclodextrin (HPBCD) (degree of substitution (DS) ~ 4.5), β-cyclodextrin (BCD), random methyl-β-cyclodextrin (RAMEB) (DS~12), and sulfobutylated β-cyclodextrin sodium salt (SBECD) (DS~4) were the product of Cyclolab Ltd. (Budapest, Hungary), chrysin (5,7-Dihydroxyflavone) was purchased from Alfa Aesar (by ThermoFisher Scientific, Kandel, Germany), and all other reagents are from Sigma.

### 2.2. Methods

#### 2.2.1. Preparation of Chrysin–Cyclodextrin Complexes

Chrysin–cyclodextrin complexes were produced by lyophilization in different molar ratios. Chrysin was dissolved in 96% ethanol with sonication at 3.33 mg/ml concentration. RAMEB and HPBCD were dissolved in this solution, and 0.4 ml of water was added to each ml of the solution. After that, the samples were frozen at −110 °C and lyophilized to evaporate the solvents with a ScanVac CoolSafe freeze dryer (Labogene, Allerød, Denmark). As SBECD and BCD are not soluble in a suitable extent in ethanol, they were dissolved in purified water and added to the chrysin solution. A slight precipitation could be observed upon mixing the components. These samples were also frozen and lyophilized. After lyophilization, yellow, solid products were obtained, which were ground in mortar and used in further experiments. Using the same complexation method, chrysin–cyclodextrin complexes with 1:1 and 1:2 molar ratios were prepared. Chrysin–BCD complexes were prepared just at a 1:1 molar ratio, due to the low solubility of BCD. The complexes were kept at −20 °C until the experiments.

#### 2.2.2. Solubility Test

The lyophilized complexes (1:1 and 1:2) were tested for the solubility improvement of chrysin and compared to the solubility of chrysin in water. For this, 2 ml of water for injection was added to 15 mg complexes, and a dispersion was made by vortexing. The mixtures were rotated in 5 ml Eppendorf tubes for 24 h at room temperature and then centrifuged at 2000 rpm for 10 min. The supernatant was collected, and the absorbance of chrysin in solution was determined by UV spectrophotometer (Shimadzu UV-1900). Chrysin concentration was calculated by the calibration curve determined in ethanol. The solubility of chrysin in water was 1.01 ± 0.07 µg/ml. The solubility increment of chrysin was calculated by dividing the solubility values of chrysin complexes by the solubility of chrysin in water.

#### 2.2.3. Phase-Solubility Test

The phase-solubility test was performed by adding a fixed excess amount of chrysin powder to 2 ml solutions containing four different kinds of cyclodextrins, βCD, RAMEB, HPBCD, and SBECD, at increasing concentrations. In the capped vials, excess amounts of chrysin powder (60 mg) were measured, in which a constant volume of distilled water (2 ml) and increasing concentration of each cyclodextrin (5–80 mM) were placed. In the case of βCD, the serial dilution was prepared at the concentration from 0.5 to 8 mM, due to the poor solubility of βCD in distilled water. The vials were vortexed for 30 seconds to achieve well-mixed dispersions. They were rotated at room temperature and protected from light. After 72 h, each vial was centrifuged at 15,000 rpm for 10 min. The samples were taken from the clear supernatant, and the chrysin content of the samples was analyzed by UV spectrophotometer (Shimadzu UV-1900). The phase solubility profiles of chrysin were achieved by plotting the solubility of chrysin versus the concentration of the cyclodextrins. The apparent stability constants (K_s_) of chrysin–CD complexes were calculated from phase-solubility diagrams according to the following equation:

(1) $$K_{s}=\;\frac{\mathrm{slope}}{S_{0}\left(1-\mathrm{slope}\right)}$$

S_0_—Chrysin solubility in water

The complexation efficiency (CE) and drug:cyclodextrin molar ratio (D:CD) were calculated as follows:

(2) $$\mathrm{CE}=\;\frac{\mathrm{slope}}{(1-\mathrm{slope})}$$

(3) $$D:\mathrm{CD}=1:(1+\frac{1}{CE})$$

#### 2.2.4. Cell Culture

Human adenocarcinoma Caco-2 intestinal epithelial cells [European Collection of Cell Cultures (ECACC, UK)] were grown routinely in Dulbecco’s Minimum Essential Medium (DMEM), which was supplemented with 10% fetal bovine serum at 37 °C in 5% CO_2_ atmosphere. 

#### 2.2.5. Cell Viability Study

For the viability studies, Caco-2 cells were seeded in 96-well plates at a density of 10^4^ cells/well, cultured until monolayer formation, and treated with increasing concentrations of cyclodextrin–chrysin complexes with different molar ratios as follows. 

The viability of Caco-2 cells was determined by using the MTT assay method. The chrysin–CD complexes of different ratios (1:1, 1:2) were dispersed in phosphate-buffered saline (PBS) at the concentration of 200 µM. Cells were treated with increasing concentrations of chrysin–CD (12.5–200 µM) complexes in PBS at 37 °C for 30 min (n = 6 for each group). The control group was processed equally and incubated without the complexes simultaneously. After 30 min of incubation, cells were washed with PBS, and the MTT solution was added to each well at a final concentration of 0.5 mg/ml. Then, cells were incubated for 4 h at 37 °C until the purple formazan crystals were formed and the crystals were dissolved in isopropanol/1 N HCl (25:1). The absorbance was measured at 570 nm with a FLUOstar OPTIMA microplate reader (BMG LABTECH, Offenburg, Germany). Absorbance values were corrected with background absorbance, which was measured at 690 nm. Cell viability was expressed as the percentage of the untreated control.

#### 2.2.6. Permeability Study on Caco-2 Monolayers

For permeability studies, cell monolayers were grown on 1.12 cm^2^ permeable Transwell^®^ polycarbonate filters with a 0.4 µm pore size (Corning, Lowell, MA, USA). Caco-2 monolayers were used for experiments after 14–21 days of initial seeding, when the transepithelial electrical resistance (TEER) reached 900 Ωcm^2^. Chrysin–CD complexes were dispersed in Hank’s Balanced Salt Solution (HBSS), diluted to get 100 µM concentration, and sterile filtered through a 0.22 µm syringe membrane filter. Cell monolayers were washed with HBSS, and the solutions of chrysin–CD complexes were put onto the apical surface of cell layers. At certain time points, samples were taken from the basal sides of the cell layers and the permeated amount of chrysin was measured by the HPLC method. The apparent permeability coefficient (P_app_) of chrysin was calculated using the following equation:

(4) $$P_{\mathrm{app}}=\frac{dQ}{dt}\cdot \frac{1}{\left(C_{0}\cdot A\right)}$$

where P_app_ is the apparent permeability coefficient (cm/s); *dQ*/*dt* is the permeability rate of substances (mol/s); *C*_0_ is the initial concentration of the substances in the upper compartment (mol/mL); and A is the surface area of the membrane (cm^2^).

#### 2.2.7. High-Performance Liquid Chromatography Method

For High-Performance Liquid Chromatography (HPLC) analysis, a Merck–Hitachi LaChrom HPLC system fitted with a diode array detector was used. The sample measurements and data evaluation were done by Ezchrom Elite software. For the separation, a C-18 (150 × 2.1 mm, particle size: 5 µm) reversed-phase column was applied. The chromatographic binary mobile phase consisted of 1% acetic acid in water (A)–methanol (B) (30–70%) at a flow rate of 1.0 ml/min. At the mentioned flow rate, the injection volume of 95 µl with a 5-min run time was performed, while the temperature of the column was maintained at 25 °C. The chromatograms were recorded at 275 nm. For the evaluation of linearity, a standard stock solution of Chrysin was prepared at 100 µg/ml in methanol, and working standards were made by diluting the stock solution with methanol from 0.5–50 µg/ml. The standard curve was constructed plotting peak area versus concentration, with a regression coefficient of 0.999.

#### 2.2.8. Statistical Analysis

For statistical analyses, SigmaStat software (version 3.1; SPSS Inc., Chicago, IL, USA) and GraphPad Prism 5.0 software (GraphPad Software Inc., La Jolla, CA, USA) were used. For figure preparation, GraphPad Prism 5.0 and ChemDraw Prime software (PerkinElmer, Waltham MA, USA) were used. Data are presented as means ± SD. Comparisons of groups were performed using ANOVA. Differences were considered significant at *p* < 0.05.

## 3. Results

### 3.1. Solubility Test

**Figure 2:**
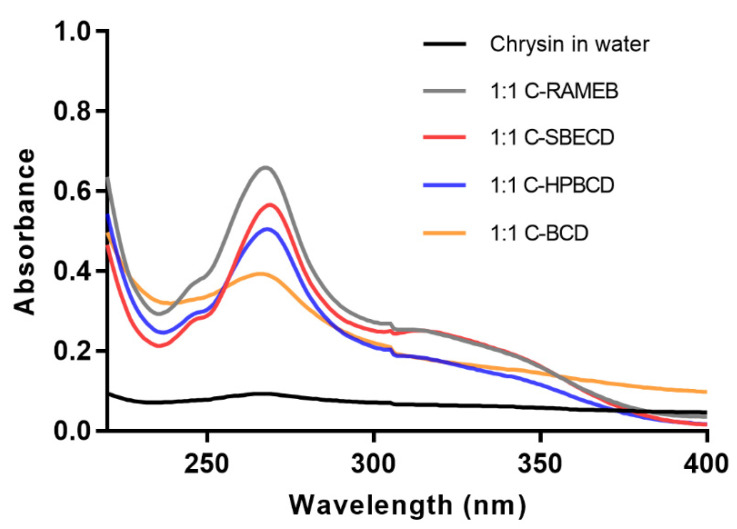
Representative UV spectra of chrysin–cyclodextrin complexes in water. Concentration values of dissolved chrysin are shown in Table 1.

The solutions of chrysin–cyclodextrin complexes (1:1 and 1:2) were measured by UV spectrophotometer. The spectra of the 1:1 chrysin:cyclodextrin complexes and chrysin dissolved in water can be seen in Figure 2. 

Chrysin–RAMEB complexes showed the highest solubility, while SBECD, HPBCD, and βCD complexes of chrysin showed lower solubility at both ratios. The measured chrysin concentrations are summarized in Table 1. The effect of cyclodextrins on chrysin solubility in water was calculated and expressed by the solubility increment of chrysin, which was compared to the measured solubility of chrysin in water (1.01 ± 0.07 µg/ml) (Table 1).

The most effective was RAMEB, the methylated derivative of beta-cyclodextrin both in 1:1 and 1:2 molar ratios, while the least effective was βCD. Interestingly, in 1:2 molar ratios, HPBCD had a higher solubilization value than SBECD. Due to the poor solubility of βCD, its 1:2 complex was not prepared and used in the experiments. 

### 3.2. Phase-Solubility Test

**Figure 3:**
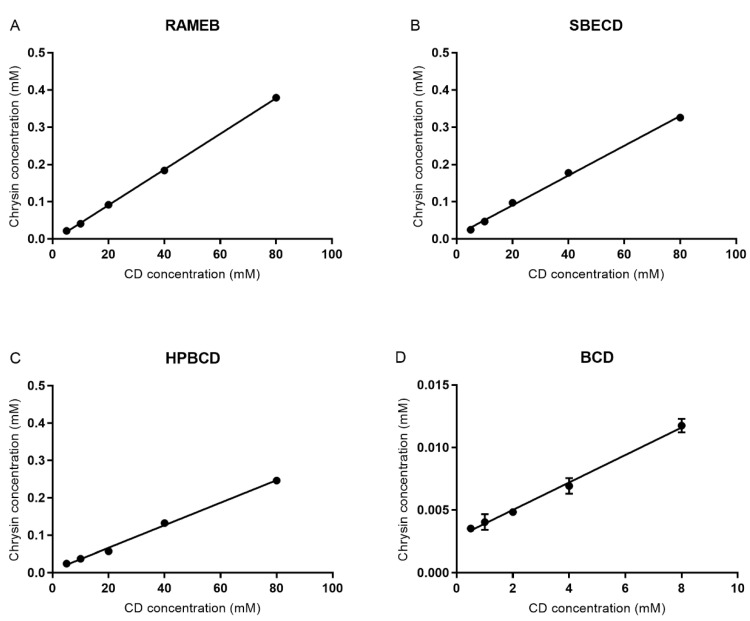
Phase-solubility diagrams of chrysin with (**A**) RAMEB, (**B**) SBECD, (**C**) HPBCD, and (**D**) βCD.

Figure 3 shows the solubility profiles of chrysin in the presence of RAMEB, SBECD, HPBCD, and βCD. Due to the limited water solubility of βCD, the highest applied concentration was 8 mM. Each cyclodextrin derivative was able to improve the water solubility of chrysin in a cyclodextrin concentration-dependent manner. 

Stability constants (K_s_) of chrysin–cyclodextrin complexes were calculated from the phase-solubility data. The strongest interaction was detected in the case of RAMEB and SBECD (Table 2.).

### 3.3. Cell Viability Study

**Figure 4:**
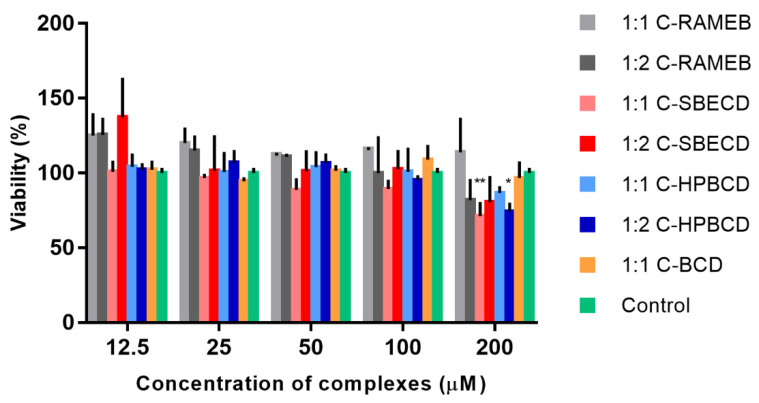
Cytotoxicity of cyclodextrin–chrysin complexes on Caco-2 cells after 30 min of incubation in the concentration range of 12.5–200 µM (data are presented as means ± SD, *p* < 0.05, n = 6).

Figure 4 shows that cyclodextrin–chrysin complexes were not cytotoxic on Caco-2 cells after 30 min of incubation up to 100 µM concentration, but at 200 µM, the cell viability decreased below 80%. Interestingly, 1:1 chrysin:SBECD and 1:2 chrysin:HPBCD complexes significantly reduced the cell viability compared to the untreated control (*p* < 0.05, n = 6). The RAMEB complex did not show cytotoxicity.

### 3.4. Permeability Study on Caco-2 Monolayers

**Figure 5:**
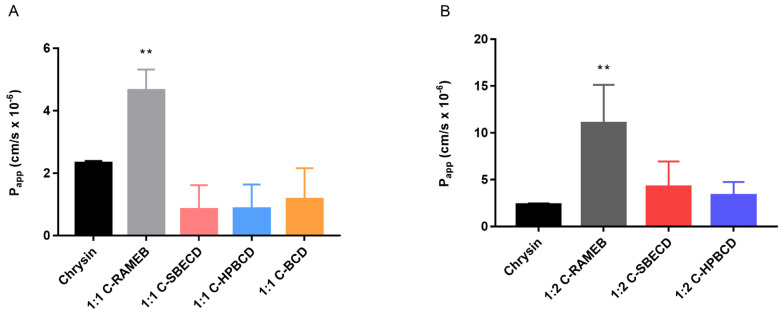
Permeability of chrysin on Caco-2 monolayers treated with 1:1 (**A**) and 1:2 (**B**) chrysin–cyclodextrin complexes. RAMEB significantly increased P_app_ of chrysin both at 1:1 and 1:2 molar ratios (data are presented as means ± SD, *p* < 0.01, n = 3) (P_app_—apparent permeability coefficient).

The permeability of chrysin using cyclodextrin complexes of 1:1 and 1:2 molar ratios were tested on Caco-2 monolayers. In the case of 1:1 and 1:2 complexes, RAMEB was the only cyclodextrin that was able to improve significantly the chrysin permeation. The P_app_ values of chrysin, 2.32 × 10^−6^ cm/s, was increased to 4.65 × 10^–6^ cm/s and 1.1 × 10^−5^ cm/s by the application of 1:1 and 1:2 RAMEB complexes on Caco-2 monolayers, respectively (Figure 5).

The 1:2 chrysin–βCD complex was not prepared due to the low solubility of βCD in water. TEER values of the Caco-2 monolayers were measured before and after the permeability experiments. No significant decreases in TEER values were observed after the 2 h chryisn–cyclodextrin treatments, indicating that the complexes did not cause damages in the integrity of the monolayers.

## 4. Discussion

The bioavailability of chrysin is inadequate due to its low water solubility and poor absorption from the gastrointestinal tract. Despite its low water solubility, just a limited number of publications can be found focusing on the solubilization of chrysin. Folate-conjugated pluronic PF127-pluronic F68 mixed micelles [15], solid dispersions with surface active agents [16], and nanoparticles [17,18] were used to improve the bioavailability and effect of chrysin. Amino-appended cyclodextrins [19], SBECD [20], and βCD [21] were also used for the complex formation and solubilization of chrysin, but interestingly, the effect of cyclodextrins on the Caco-2 intestinal permeability of chrysin has not been studied yet. Our aim was to prepare chrysin–cyclodextrin complexes with different cyclodextrin derivatives, such as BCD, RAMEB, SBECD, and HPBCD in 1:1 and 1:2 molar ratios and test their chrysin permeability enhancement on Caco-2 cells. At first, we prepared the complexes by lyophilization and tested the solubility of complexed chrysin. RAMEB was the most effective CD derivative, as we found a 7.41 times solubility increase in the case of the 1:1 chrysin:RAMEB complex compared to the solubility of chrysin in water. The higher molar ratio (1:2) resulted in 8.04 times solubility improvement. Both SBECD and HPBCD showed lower solubilization values (6.29 and 5.66, respectively), and βCD had the lowest solubilization effect (4.37 times solubility improvement) in the case of 1:1 complexes. We have to mention that only the 1:1 chrysin:βCD complex was prepared due to the poor solubility of βCD in the applied solvents. Other studies found a much higher solubilization of chrysin by SBECD [20] and amino-appended βCD [19], but it should be noted that the published chrysin water solubility varied greatly according to the different sources [19]. The phase-solubility tests confirmed the solubilization results. All of the CD derivatives were able to solubilize chrysin in water and had linear, A_L_-type phase solubility curves in the function of cyclodextrin concentration, assuming a 1:1 binding stoichiometry. RAMEB and SBECD showed the highest apparent stability constants, 1200 M^−1^ and 1000 M^−1^ respectively, while HPBCD and BCD had weaker interactions. Other studies showed higher constant values, but the tendency was the same for SBECD, HPBCD, and BCD [20]. It is important to note that the K_s_ values are highly influenced by the measured water solubility of chrysin (S_0_). The observed differences between the presented and earlier results can be also explained by the possible variance of the different water solubility values of chrysin. To avoid this discrepancy, we calculated the complexation efficiency (CE) and the drug:cyclodextrin molar ratio (D:CD) from the slope of the phase-solubility profiles [22]. CE and D:CD values were low, indicating that the majority of cyclodextrin molecules are free in the chrysin–cyclodextrin solution. Apparently, low CE and D:CD values are inconsistent with the K_s_ values. It can be explained by the fact that cyclodextrins form both inclusion and non-inclusion complexes, and different types of complexes can coexist in aqueous solutions [22]. In this case, it indicates that chrysin forms also non-inclusion complexes and forms molecular aggregates with cyclodextrin, which take part in the solubilization of chrysin. 

The structure of the cyclodextrin derivatives also influences the chrysin–cyclodextrin interaction. The crystalline natural β-cyclodextrin has limited solubility in water, which limits its solubilization ability. The random substitution of the cyclodextrin ring results in an amorphous product with increased solubility for the cyclodextrins themselves as well as for their complexes. Methylated derivatives containing lipophilic methoxy moieties (such as RAMEB) or hydroxypropylated (HPBCD) and sulfobutylated (SBECD) hydrophilic derivatives have higher solubility and solubilization properties [23]. On the other hand, the molecular configuration of the cyclodextrin torus influences also the interactions with the guest molecule. H3 and H5 hydrogens attached to carbon 3 and 5 of the component glucose residues are located in the cyclodextrin cavity and sensitive to the molecular interactions [24]. An earlier study reported strong hydrogen bonding potential between the proton of OH-5 and proton of OH-7 of chrysin and the H3 and H5 of SBECD, respectively. The H3 proton can also interact with the C = O group of chrysin [20]. Interestingly, in this configuration, the aromatic ring of chrysin is situated outside or near to the secondary face of the cyclodextrin ring. Nevertheless, it can explain the stronger interaction with RAMEB, which has lipophilic methyl groups on the cyclodextrin ring.

Interestingly, no previous data can be found for RAMEB applying in phase-solubility studies of chrysin. In another work, with the flavone glycoside baicalin, where RAMEB was involved in the experiments, a similar order was reported for the stability constants: RAMEB > SBECD > HPBCD > βCD [25].

The biocompatibility of chrysin–cyclodextrin complexes were tested on Caco-2 cells before the permeability test. No significant cytotoxicity was detected up to 100 µM, while at 200 µM, the cell viability decreased significantly in the case of some complexes (Figure 4.). In the following permeability tests, this limitation was considered. The Caco-2 permeability test of chrysin complexes revealed that chrysin:RAMEB complexes were the most effective to improve the chrysin permeability through the Caco-2 monolayers. The P_app_ values of native chrysin (2.32 × 10^−6^ cm/s) are comparable to an earlier study (2.6 × 10^−6^ cm/s) testing polyphenols on Caco-2 monolayers [14]. Both the 1:1 and 2:1 chrysin:RAMEB complexes increased significantly the permeability of chrysin through the Caco-2 monolayers, while the effect of the other cyclodextrin derivatives were not significant compared to chrysin permeability (Figure 5.) RAMEB is a lipophilic cyclodextrin derivative with several effects on cell membranes and monolayers, which can influence drug permeability. The solubility and permeability enhancement of drugs through the unstirred water layer (UWL) can be the major mechanisms, but cell membrane permeabilization, changes in the tight junctions (TJ), and endocytosis of the complexes could be also mentioned [26]. It was found earlier that the basolateral to apical flux of chrysin was about 2-fold higher than the apical to basolateral flux on Caco-2 monolayers. Verapamil was without an inhibitory effect; thus it was concluded that this mechanism did not appear to involve P-glycoprotein. Walle et al. revealed the efflux of glucuronic acid and a sulfate conjugate; thus, their observations with the efflux of chrysin metabolites in the Caco-2 cells suggested that multidrug resistance associated protein 2 (MRP2) was the transporter. They concluded that the metabolism by the Caco-2 cells may limit the oral bioavailability of chrysin [13]. From this point of view, the most probable mechanisms by which RAMEB increases chrysin permeability are the solubility improvement and permeability enhancement through the UWL. TEER values did not decrease after the experiment; thus, changes in TJ structures can be excluded. We detected TJ and TEER alterations caused by RAMEB at much higher concentrations (20 mM) earlier [27]. Nevertheless, the endocytosis cannot be excluded, as we reported earlier by the fluorescent paclitaxel derivative, Flutax–RAMEB complexes [28]. Even if the effects of cyclodextrins were not tested on the flavone chrysin permeability, other isoflavones such as genistein and daidzein were complexed and studied on Caco-2 monolayers. HPBCD and RAMEB had better solubility enhancement on genistein and daidzein than βCD and gamma-CD (γCD), and RAMEB was the most effective to improve the permeability of daidzein [29]. Although the chemical structures of these molecules are similar, the position of the B-ring and the number and position of hydroxyl groups influence their host–guest interaction with cyclodextrins. According to the data, RAMEB and SBECD are suitable cyclodextrin derivatives for the chrysin complexation, and especially RAMEB for the permeability enhancement of chrysin and other flavones. 

## 5. Conclusions

In conclusion, we successfully prepared and characterized chrysin–cyclodextrin complexes. The solubility and permeability of chrysin could be improved by RAMEB, SBECD, HPBCD, and BCD, but among these CD derivatives, RAMEB was the most effective both in solubilization and permeability enhancement. Between 12.5 and 100 µM concentration, cyclodextrin-hrysin complexes were not cytotoxic on Caco-2 cells, and interestingly, the RAMEB complex did not show cytotoxicity. These complexes can be further tested in vitro and in vivo for their biological activities.

## Figures and Tables

**Table 1:** Concentration values and solubility increment of chrysin in water by cyclodextrin complexation (n.d.—not determined) (n = 3). βCD: β-cyclodextrin, HPBCD: hydroxypropyl-β-cyclodextrin, RAMEB: randomly-methylated-β-cyclodextrin, SBECD: Sulfobutylether-β-cyclodextrin.

	Chrysin in H_2_O	Chrysin:RAMEB		Chrysin:SBECD		Chrysin:HPBCD		Chrysin: βCD	
Complex molar ratio	-	1:1	1:2	1:1	1:2	1:1	1:2	1:1	1:2
Chrysin conc. (ug/ml)	1.01 ± 0.07	7.48 ± 0.15	8.12 ± 0.42	6.35 ± 0.11	7.32 ± 0.2	5.72 ± 0.28	7.59 ± 0.17	4.42 ± 0.37	n.d.
Solubility increment	1	7.41	8.04	6.29	7.25	5.66	7.52	4.37	n.d.

**Table 2:** Stability constants (K_s_) complexation efficiency (CE) and the drug:cyclodextrin molar ratio (D:CD) of chrysin–cyclodextrin complexes (n = 3).

Cyclodextrins	K_s_ (M^−1^)	CE	D:CD
RAMEB	1200	0.0048 ± 6.9 × 10^−5^	1:209 ± 3
SBECD	1000	0.004 ± 2.1 × 10^−5^	1:250 ± 4
HPBCD	760	0.003 ± 7 × 10^−5^	1:332 ± 2
βCD	275	0.0011 ± 5.9 × 10^−5^	1:912 ± 49
